# Detection of Nonhematologic Neoplasms in Bone Marrow by Flow Cytometry: A Report of Two Cases

**DOI:** 10.7759/cureus.51414

**Published:** 2023-12-31

**Authors:** Athena Myrou

**Affiliations:** 1 Department of Internal Medicine, American Hellenic Educational Progressive Association (AHEPA) University Hospital, Thessaloniki, GRC

**Keywords:** neuroendocrine neoplasms, bone marrow, non-hematopoietic malignancies, solid tumors, multiparameter flow cytometry

## Abstract

Multiparameter flow cytometry (MFC) is a well-established method for the diagnosis, prognosis, and follow-up of a vast majority of hematological malignancies; however, it can have a major impact on the rapid diagnosis of nonhematopoietic tumor micrometastases in minimally invasive samples such as bone marrow aspirates (BMAs), body fluids, and tissue samples (lymph nodes, fine needle aspirates).

Here, we present two cases of bone marrow micrometastases of neuroendocrine origin (one small cell lung carcinoma [SCLC] and one large cell neuroendocrine carcinoma [LCNEC] of the lungs) readily recognized by routine MFC investigation of BMA and review the existing literature on the role of MFC in the diagnosis of solid tumors of neuroendocrine origin.

The clinical application of flow cytometry for the diagnosis of solid tumors is limited despite the accumulating evidence of the value of the method. It can be of great value in situations where the patient’s clinical status forbids invasive procedures, and a rapid diagnosis is desirable.

Flow cytometry is a valuable tool for the detection of both hematological and nonhematologic neoplasms. Future large-scale patient series will probably confirm its role in the screening, diagnosis, and classification of more tumor types.

## Introduction

Multiparameter flow cytometry (MFC) is a well-established method for the diagnosis, prognosis, and follow-up of a vast majority of hematological malignancies and can provide, rapidly and effectively, information about the simultaneous expression of multiple proteins/markers, on the surface or intracellularly, of thousands to millions of single cells. It can be performed in any single cell suspension such as peripheral blood (PB), bone marrow aspirate (BMA), or other body fluids, as well as in tissue samples (e.g., lymph nodes, fine needle aspirates), after proper processing. However, MFC’s role in the diagnosis of solid tumors and nonhematopoietic malignancies is under evaluation, as it requires specially designed protocols (sample preparation, antibody panels, gating strategy), and therefore it is not routinely applied in clinical flow cytometry laboratories [1].

In a range of examined specimens, including BMAs, nonhematopoietic malignant cells were found to reside within the CD45-negative populations. Commonly, these populations exhibit morphological features of the so-called small round cell tumors or small round blue cell tumors (SRBCTs), a heterogeneous group of neoplasms composed of relatively small, round to oval, closely packed undifferentiated cells with high nuclear-to-cytoplasmic ratio, scant cytoplasm, and round nuclei with evenly distributed, slightly coarse chromatin and small or inconspicuous nucleoli [2]. The blue coloration of the cells is indicative of their large hyperchromatic nuclei and thin, basophilic cytoplasmic rim [3]. Despite a similar light microscopic morphology, SRBCTs include pathologic entities from vastly different lineages, including (1) epithelial tumors such as small cell carcinoma (SCC) (poorly differentiated neuroendocrine carcinoma); (2) mesenchymal tumors encompassing malignant solid neoplasms of childhood and other small round cell sarcomas; and (3) tumors with overlapping features, such as lymphoma and melanoma [3]. The diagnosis of these tumors represents a challenge for cytopathologists, and the complexity, herein, stems from the fact that these tumors share not only similar morphological features but also some immunophenotypic characteristics, thus requiring a broad panel of antibodies, not included in basic immunohistochemistry panels, commonly used in the routine work of most pathology laboratories [4].

Here, we present two cases of lung carcinomas of neuroendocrine origin with bone marrow micrometastases (one small cell lung carcinoma [SCLC] and one large cell neuroendocrine carcinoma [LCNEC] of the lung) detected by flow cytometry analysis of BMA and review of the existing literature on the role of flow cytometry in the diagnosis of solid tumors of neuroendocrine origin.

## Case presentation

Case 1

A 67-year-old woman was admitted to a rural hospital due to dyspnea, precordial pain, paroxysmal atrial fibrillation (PAF), and lower limb edemas. During her hospitalization, heart rate control was achieved (two episodes of PAF) and excessive hypokalemia (serum potassium (K+) level of 1.85 mEq/L) was corrected. Yet, computed tomography (CT) showed findings relevant to left adrenal gland tumor. Thus, the patient was referred to our hospital for further investigation.

On admission, the woman presented with congestive heart failure symptoms and findings, PAF on electrocardiographic examination (ECG), and abdominal ecchymoses. Laboratory testing revealed K+ level of 2.5 mEq/L, blood glucose level of 648 mg/dL, platelet (PLT) count of 100K/dL, hematocrit (Hct) of 25.8%, and metabolic alkalosis (pH of 7.53, PaCO_2_ of 35 mmHg, HCO_3_ of 29 mEq/L, ketones of 1.2 mmol/L). Glycated hemoglobin (HbA1c) was 9.2%, and diabetes mellitus type 2 was added to her diagnoses. Though her hemodynamic, metabolic, and respiratory status gradually improved, anemia and thrombocytopenia worsened. Multiple daily transfusions with red blood cells (RBCs) and PLTs were needed, and after hematological consultation, therapy with dexamethasone and immunoglobulin was initiated. Along with the latter, additional CT imaging revealed left lower lung tumor, mediastinal lymphadenopathy, a mass in the pancreas body (3.9 x 2.3 cm), and abnormal soft tissue below the pancreatic neck and behind the duodenum.

In PB and BMA smears stained by May-Grunwald Giemsa, abnormal blast-like cells were observed comprising 3% and 8% of total nucleated cells, respectively.

**Figure 1:**
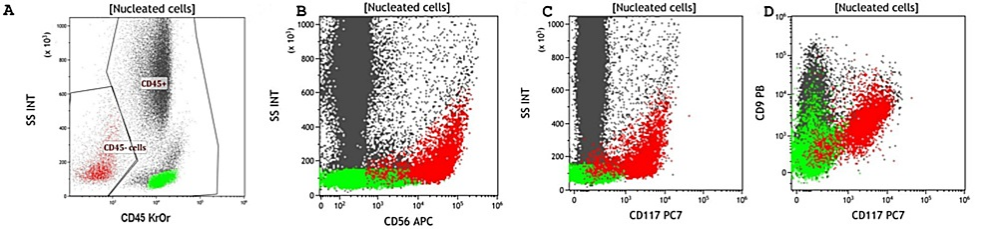
Representative flow cytometry dot plots of the abnormal population infiltrating bone marrow. Small lung cell carcinoma cells are typically negative for CD45 (A) and positive for CD56 (B), CD117 (C), and CD9 (D). Red dots indicate tumor cells, and green dots indicate lymphocytes.

MFC analysis of BMΑ demonstrated an abnormal population within the CD45 negative region, expressing strongly CD117, CD56, CD24, CD9, and CD15, and weakly CD10 and CD71, whereas there was no significant expression of other hematopoietic markers analyzed (CD19, CD22, cCD3, CD7, CD13, CD33, CD65, CD64, CD14, CD38, CD138, CD36, cCD61, cCD41, CD34, HLADR, cTdT and cMPO) (Figure 1).

**Figure 2:**
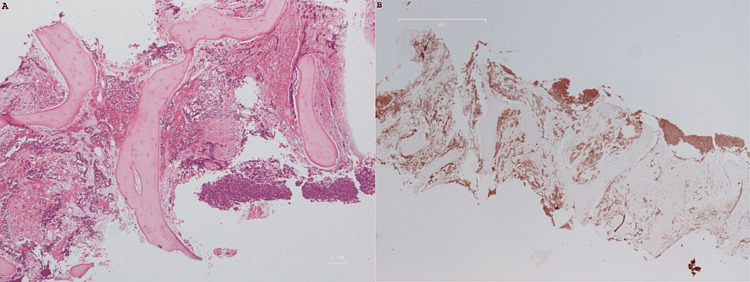
Bone marrow biopsy. (A) Bone marrow spaces infiltrated by tightly packed neoplastic cells (H&E). (B) Neoplastic cells are positive on immunohistochemical stain for synaptophysin bone marrow spaces. H&E, hematoxylin-eosin stain

Further pathological examination of bone marrow biopsy revealed infiltration of medullary spaces by aggregates of neoplastic cells, tightly packed, with a relatively uniform appearance. The cells were small, with round nuclei and scant cytoplasm, and exhibited the following immunophenotypes: CD56+, synaptophysin+, chromogranin++/-, CK8/18+, TTF1+, CK7++/-, CD20-, CD3-, MUM1-, MPO-, elastase-, glycophorin-, and CDX2- (Figure 2). The immunohistochemical and morphological results were consistent with the presence of neuroendocrine neoplasm that had invaded the bone marrow and exhibited features of small cell lung carcinoma likely originating from the lungs.

Unfortunately, 20 days after her admission, the patient suffered from an intracerebral hemorrhage and died seven days later in the intensive care unit.

Case 2

**Figure 3:**
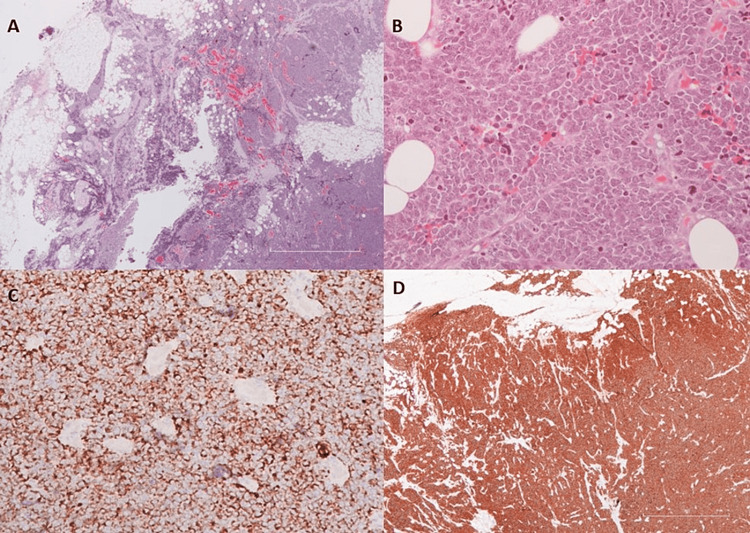
Cervical lymph node biopsy. (A) Lymph node neoplastic infiltration by large cell neuroendocrine carcinoma at low magnification presenting mainly with syncytial growth pattern. At the left of the picture, the tumor is arranged in trabeculae and ribbons of neoplastic cells. (B) High power view of large cells with syncytial growth pattern, marked nuclear atypia with large at some degree rounded nuclei, prominent nucleoli, increased mitotic activity, and moderate amphiphilic cytoplasm. These features are compatible with LCNEC. (C) Regionally moderate CK8/18 cytoplasmic immunohistochemical expression of the neoplastic cells with perinuclear accentuation. (D) Cervical lymph nodes with loss of architectural structure due to diffuse infiltration of large neoplastic cells positive for CD56. LCNEC, large cell neuroendocrine carcinoma

A 68-year-old man was referred to our hospital for generalized lymphadenopathy investigation. His past medical history involved only arterial hypertension, while allergies and family history were unremarkable. The patient had undergone percutaneous cervical biopsy in a rural hospital seven days before admission. Cervical lymph node samples were completely infiltrated by malignant neoplastic lesions. Malignant cells exhibited the following immunophenotype: CK8/18+, CD56+, Synaptophysin+, NSE+, CD99+, BCL2+, TTF1+ (gene 8G7G3/1), CD79a-, CD20-, PAX5-, CD3-, CD10-, cyclin D1-, CD23-, CD30-, Tdt-, CK7-, CK20-, NF-, chromogranin-, myogenin-, and desmin-. Morphological and immunohistochemical findings were consistent with a large cell neuroendocrine lung neoplasm (LCNEC) (Figure 3). Chest X-ray revealed infiltrations and pachypleuritic elements. Chest CT assessment confirmed chest X-ray findings (pleural effusions and generalized lymphadenopathy were noticed). The same generalized lymphadenopathy was also found on abdominal CT.

**Figure 4:**
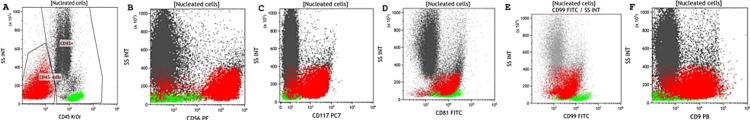
Representative flow cytometry dot plots of bone marrow aspirate Large cell neuroendocrine carcinoma is characterized by the absence of CD45 expression (A) and the presence of CD56 (B), CD117 (C), CD81 (D), CD99 (E), and CD9 (F) expression. Red dots indicate tumor cells, and green dots indicate lymphocytes.

Due to generalized lymphadenopathy, BMA was sent for MFC immunophenotypic analysis, which revealed the presence of a CD45-/CD56+ population comprising 28% of the nucleated cells of the aspirate with further immunophenotypic characteristics the expression of CD117, CD9, CD58, CD99, CD81 and CD57, and no other significant expression of hematopoietic markers (CD19, CD22, cCD3, CD7, CD33, CD65, CD64, CD14, CD38, CD138, CD36, cCD61, cCD41, CD34, HLADR, cTdT and cMPO) (Figure 4).

This finding was consequently confirmed by bone marrow biopsy, which demonstrated infiltration at a rate of 5% by the same neuroendocrine neoplasm.

Unfortunately, soon after admission, the patient’s clinical status deteriorated due to progressive respiratory distress. A pleural puncture was attempted by a thoracic surgeon to evacuate pleural effusion; yet, during the procedure, the patient suffered a cardiac arrest and died.

## Discussion

Malignant tumor diagnosis and classification is traditionally based on the histopathological/cytomorphological evaluation of tissue specimens or body fluids. Although histopathology/cytomorphology is considered the gold standard, diagnosis can be challenging, especially in cases of SRBCTs where morphological and immunohistochemical similarities may be quite confusing. Additionally, many patients with malignancy, particularly lung cancer, often present in advanced stages of disease, and surgical resection of the primary lesion with curative intent is usually not a feasible option for testing [5]. For those patients, minimally invasive procedures may be implemented for obtaining a cytology specimen for traditional histopathological/cytomorphological examination, sometimes assisted by ancillary techniques, such as immunochemistry, karyotyping, fluorescence in situ hybridization (FISH), and molecular studies, which are all time-consuming and laborious [5].

MFC can be an appealing alternative in such cases, providing a rapid diagnosis especially in emergency situations or when a patient’s life is threatened (e.g., superior vena cava syndrome). Additionally, MFC routine immunophenotypic panels for ΒΜΑ, fluid, and tissue samples can promptly identify infiltrating nonhematologic malignancies even in the absence of more elaborate markers [6] used in specific solid tumor diagnostic panels, such as epithelial cell adhesion antigen (EpCAM or CD326), cytokeratin 5/8 (CK 5/8), epithelial mucin (MUC-1), cytokeratin 18 (CK18), nuclear (nu)MYOD1, (nu)myogenin, GD2, CD90, CD271, NG2, and others [7-9].

Lung neuroendocrine tumors account for approximately 20% of all primary lung cancers [10]. Pulmonary LCNEC is a rare but highly aggressive tumor with neuroendocrine differentiation, representing around 3% of all lung cancers. Prognosis is poor with life expectancy being between 8 and 12 months [8]. According to Quiros-Caso et al. [9], neuroendocrine tumors show an immunophenotypically profile, which clearly differentiates them from other neoplasms, mainly due to the expression of CD56 (90% of the cases) along with the expression of CD117 (61%), CD326 (97.5%), CD81 (93.4%), and CD9 (53%) in combination with negative expression for CD38, CD3, CD19, CD20, CD4, CD8, and CD45 (sometimes dimly expressed).

The neural cell adhesion molecule (NCAM) CD56 is a member of the immunoglobulin superfamily and is involved in cell adhesion regulating mechanisms, intracellular signaling, and cytoskeleton dynamics [11]. It is normally expressed on neuroendocrine tissues, natural killer (NK) cells, and a subset of T cells and monocytes. Among hematological malignancies, CD56 expression has been described in those of T/NK derivation, multiple myeloma, acute myeloid leukemia, myelodysplastic syndromes, and myeloproliferative disorders. CD56 expression has been reported to predict a higher risk for central nervous system disease in acute lymphoblastic leukemia. Moreover, CD56 is the most detected marker by MFC in nonhematopoietic malignancies of neuroendocrine origin [6,9,12].

Light scatter characteristics (increased forward and side scatter) of an abnormal population combined with the absence of CD45 and other hematopoietic lineage specific marker expression, together with the expression of CD56 and other commonly used markers for routine screening of BMAs, may raise the suspicion of the solid tumor bone marrow micrometastases. Clinical examination and histopathological analysis of the patient can be better focused on within a few hours to arrive at a more precise diagnosis.

MFC is a powerful diagnostic tool with the advantages of automation, reproducibility, speed (the results can be available within hours), and a high degree of objectivity (antigen expression can be quantifiable) and sensitivity. It is widely used in the diagnosis and classification of hematologic malignancies, while several nonmalignant or premalignant hematological disorders may benefit from this technology in clinical laboratories [13]. However, the clinical application of flow cytometry for the diagnosis of solid tumors is limited despite the accumulating evidence of the value of the method [6-9,14,15].

It can be of great value in situations where the patient’s clinical status forbids invasive procedures or when a rapid diagnosis is desirable [12] if the appropriate specimen is provided to the clinical flow laboratory. Finally, there are promising results of using automated flow cytometry and machine learning in the early detection of lung carcinomas, including cases with nodes <20 mm in diameter [16].

## Conclusions

Flow cytometry serves as a valuable instrument for identifying both hematological and nonhematologic neoplasms. However, its application in nonhematological neoplasms lacks established integration into routine clinical practice. Pathologists confront the difficulty of considering the likelihood of a CD45-negative population in the BMA, which suggests the presence of nonhematopoietic neoplasms (solid tumors), and distinguishing it from hematopoietic tissue using specific markers. The inclusion of CD56 expression (neural-cell adhesion molecule, NCAM) in initial screening cytometry panels proves advantageous for detecting neuroendocrine-origin tumors, such as SCLC and neuroblastoma. The significance of MFC analysis is underscored by our documentation of two cases involving patients with SCLC and LCNEC, wherein flow cytometry successfully identified neoplastic cells infiltrating the bone marrow. The prospect of its broader application to screen, diagnose, and classify various tumor types awaits validation through larger patient series, potentially enhancing therapeutic strategies tailored to each case. Building on these cases, our aim is to ascertain the role of flow cytometry in the comprehensive screening, diagnosis, and classification of diverse tumor types, thereby facilitating judicious therapeutic decision-making.
